# Assessment of Six-Minute Walk Test Among Discharge-Ready Severe COVID-19 Patients: A Cross-Sectional Study

**DOI:** 10.7759/cureus.25108

**Published:** 2022-05-18

**Authors:** Uday Yanamandra, Puneet Saxena, Rajagopal Srinath, Anuradha Sawant, Anurag Singh, Nupur Aggarwal, Bareedu Pavan, Gayatri Duhan, Bhavya Aggarwal, Praneet Kaur

**Affiliations:** 1 Internal Medicine, Armed Forces Medical College, Pune, IND; 2 Pulmonology, Army Hospital Research and Referral (R&R), New Delhi, IND; 3 Obstetrics and Gynecology, Armed Forces Medical College, Pune, IND; 4 General Practice, Armed Forces Medical Services, New Delhi, IND; 5 Dentistry, Armed Forces Dental Services, Pune, IND; 6 General Practice, Armed Forces Medical Services, Karwar, IND

**Keywords:** c-reactive protein (crp), interleukin (il)-6, lactate dehydrogenase (ldh), inflammatory mediators, discharge criteria, 6-min walk test, coronavirus disease 2019

## Abstract

Background

Among patients hospitalized for severe pneumonia due to coronavirus disease (COVID-19), clinical stability and normal resting peripheral oxygen saturation (SpO_2_) levels are widely used as a discharge criterion after recovery. It is unknown whether a test to assess the functional exercise capacity, like a six-minute walk test (6MWT), can add to the appropriateness of discharge criteria.

Methods

A cross-sectional study was conducted at a tertiary care COVID-19 hospital in India from 01^st^ to 31^st^ May 2021. All patients considered fit for discharge after recovery from "severe" COVID-19 pneumonia were subjected to 6MWT. Fitness for discharge was assessed by clinical stability and resting SpO_2_ above 93% for three consecutive days. Patients were considered to have failed the 6MWT if there was ≥4% fall in SpO_2_ or if they could not complete the test. Serum samples were analyzed for levels of C-reactive protein (CRP), interleukin-6 (IL-6), and lactate dehydrogenase (LDH) at the time of discharge.

Results

Fifty-three discharge-ready patients with a mean age of 54.54 ± 14.35 years with a male preponderance (60.38%) were analyzed. Thirty-three (62.26%) patients failed the 6MWT with a median six-minute walk distance (6MWD) of 270 m (60-360). A total of 45 (84.91%) patients had a fall in SpO_2_ during the test. The median change in SpO_2_ (∆SpO_2_) was 5% ranging from -6% to 8%. Serum LDH was significantly higher among patients who failed the 6MWT with a median LDH of 334 IU/L (38.96-2339) versus 261 IU/L (49.2-494) (p = 0.02). The difference was not significant for CRP or IL-6. There was no statistically significant correlation between the inflammatory markers with either 6MWD or (∆SpO_2_).

Conclusion

Two-thirds of the patients considered fit for discharge after recovery from severe COVID-19 pneumonia failed 6MWT, implying reduced functional exercise capacity and exertional hypoxia. Serum LDH levels were higher in these patients but not in other inflammatory markers. None of the inflammatory markers at discharge correlated with 6MWD or ∆SpO_2_ of 6MWT.

## Introduction

India experienced the most vicious phase of the coronavirus disease (COVID-19) pandemic from March to May 2021, with a massive upsurge in the number of cases and fatalities. With over 26 million confirmed cases and deaths surpassing 274,000, the healthcare system in the country was put under unprecedented strain [1]. It became imperative to introduce effective triaging, admission, and discharge criteria to grapple with the challenge. Clinical stability and normoxemia at rest were used as criteria to discharge patients after severe COVID-19 illness. Exertional tests have previously been proposed and evaluated to predict severe COVID-19 pneumonia to guide early admission [2-5]. Based on their experience with 26 discharge-ready COVID-19 patients, Fuglebjerj et al. had proposed that the six-minute walk test (6MWT) could be used potentially as a tool for the assessment of patients before discharge [6]. Elevated levels of laboratory markers such as d-dimer, interleukin-6 (IL-6), and IL-10, and lower levels of peripheral lymphocytes at discharge may be associated with poor outcomes [7,8]. We have analyzed the results of 6MWT among the patients fit for discharge as per the prevailing national guidelines and compared it with the laboratory markers of disease severity at discharge.

## Materials and methods

Study design

We conducted a cross-sectional study at a COVID-19 care center in Western India from 1^st^ to 31^st^ May 2021. The hospital was mandated to provide in-hospital care to reverse transcriptase-polymerase chain reaction (RT-PCR)-positive severe-COVID-19 cases. Severity was defined by peripheral oxygen saturation (SpO_2_) at room air of <90% or respiratory rate of >30 breaths per minute at initial presentation. The Institutional Ethics Committee of Army Hospital Research and Referral (R&R) approved the study (approval number 07/2022), and informed consent was obtained from all enrolled patients. The study was done in accordance with the Declaration of Helsinki.

Patients

All consecutive patients planned to be discharged after 1^st^ May were enrolled in the study. Patients were considered fit for discharge if they had attained clinical stability and had resting SpO_2_ > 93% on three consecutive days at room air, based on the prevailing national guidelines. Such patients were subjected to 6MWT, in accordance with current accepted standards and protocol [9,10]. The total distance walked and the change in peripheral oxygen saturation and pulse rate from beginning to the end of the test were recorded. Any patient with hemodynamic instability, recent acute coronary syndrome or angina, acute pulmonary embolism, acute myocarditis, or any other condition that, in the attending physician's opinion, could affect the exercise performance or get aggravated by 6MWT were excluded from the study [10]. Also, individuals with baseline SpO_2_ < 94% (due to a pre-existing cardio-pulmonary illness) or unwilling to participate were excluded from the study [3]. Those individuals who were unwilling to get the inflammatory markers (i.e., IL-6, C-reactive protein [CRP], and lactate dehydrogenase [LDH]) evaluated and those whose tests could not be done within 24 hours of the 6MWT were also excluded. Between 1^st^ May and 31^st^ May 2021, 53 patients who were found fit for discharge as per the prevailing guidelines and had no contraindications to perform 6MWT were included in the study.

Methodology

Baseline pulse and SpO_2_ were recorded for all the patients. Patients were requested to walk at their own pace in a hallway of length 30 m with marks at every 1 m under strict medical supervision. SpO_2_ was monitored continuously during the test with the help of a portable pulse oximeter. The test was stopped prematurely if SpO_2_ dropped below 90% [3]. Other reasons for premature termination of the test included intolerable dyspnoea, fatigue, chest pain, diaphoresis, or uneasiness, as assessed by the attending physician. SpO_2_, pulse, distance, and time walked were recorded at the end of the test. The patients were considered to have failed the 6MWT if they had a fall in SpO_2_ ≥ 4% or if the test was prematurely terminated. The study population was also subjected to IL-6, CRP, and LDH assessment before 6MWT within a maximum time gap of 24 hours.

Statistical analysis

The findings were analyzed using the John's Macintosh Project (JMP) 16.1 (Cary, North Carolina). The continuous data were assessed for normal distribution using the Shapiro-Wilk test. Variables with normal distribution were represented as mean ± standard deviation (SD) and assessed using parametric tests, i.e., the student’s t-test. The variables without normal distribution were described as median (range) and evaluated using nonparametric tests, i.e., the Wilcoxon test. The p < 0.05 was considered significant. The correlation between the continuous variables was analyzed using logistic fit (Fit Y by X).

## Results

**Figure 1:**
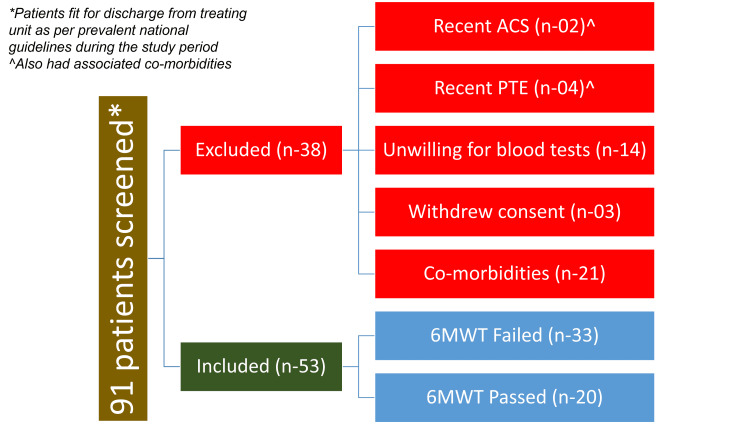
Consort diagram ACS: Acute coronary syndrome; PTE: Pulmonary thrombo-embolism; 6MWT: Six-minute walk test.

Out of 91 patients screened, 53 were analyzed (Figure 1).

The mean age of the study population was 54.54 ± 14.35 years, with a male preponderance (60.38%). Of all the patients, 33 (62.26%) failed the 6MWT test. A total of 45 (84.91%) patients had a fall in SpO_2_ during the 6MWT. The median change in SpO_2_ (∆SpO_2_) was 5% (-6 to 8%), with the mean six-minute walk distance (6MWD) being 250.36 ± 91.89 m. The characteristics of the study population, results of the 6MWT, and values of the inflammatory markers at the time of discharge are detailed in Table 1.

**Table 1:** Characteristics of the study population, results of the six-minute walk test, and values of the inflammatory markers at the time of discharge CRP: C-reactive protein; LDH: Lactate dehydrogenase; IL-6: Interleukin-6.

Characteristics	Median (Range)	Mean ± SD
Age (years)	57 (28-85)	54.54 ± 14.35
Days of hospitalization	10 (3-26)	11.94 ± 5.96
Days on oxygen	7 (0-24)	8.13 ± 5.97
Days off oxygen	4 (2-9)	3.81 ± 1.46
Distance walked	270 (60-360)	250.36 ± 91.89
Time walked	6 (1-6)	5 ± 1.62
SpO_2_ fall (%)	5 (-6-8)	4.15 ± 3.07
Pulse at the start of the test (per minute)	98 (55-160)	99.05 ± 21.19
Pulse at the end of the test (per minute)	118 (67-170)	114.56 ± 20.3
CRP at discharge (mg/L)	20 (0.5-142)	30.00 ± 30.68
LDH at discharge (IU/L)	300 (38.96-2339)	374.48 ± 329.45
IL-6 at discharge (ng/dL)	3.09 (0.25-55.89)	7.92 ± 10.71

Correlation of inflammatory markers at discharge with 6MWT results

**Figure 2:**
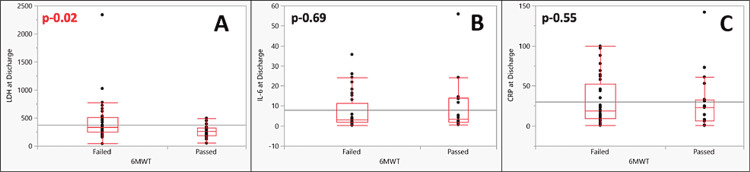
Box and whisker plot curve showing the levels of various inflammatory markers at discharge: (A) LDH, (B) IL-6, and (C) CRP among the patients who failed and passed the six-minute walk test 6MWT: Six-minute walk test; LDH: Lactate dehydrogenase; IL-6: Interleukin-6; CRP: C-reactive protein.

Patients who failed 6MWT (n = 33) had median LDH of 334 IU/L (38.96-2339), IL-6 of 3.09 ng/dL (0.25-35.65), and CRP of 18.4 mg/L (0.6-99.6) compared to 261 IU/L (49.2-494), 3.43 ng/dL (0.6-55.89) and 23 mg/L (0.5-142), respectively, for those who passed 6MWT. The difference was significant only for LDH (p-0.0203) (Figure 2).

**Figure 3:**
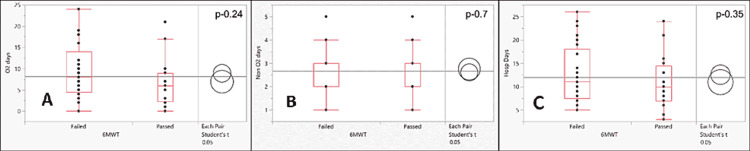
Box and whisker plot curve showing the hospitalization characteristics: (A) O2 days, (B) non-O2 days, and (C) hospitalization days among the patients who failed and passed 6MWT 6MWT: Six-minute walk test; O2 days: Number of days on oxygen; non-O2 days: Number of days off oxygen; Hosp days: Number of days in the hospital.

The comparison for days of hospitalization among the two groups is depicted in Figure 3.

**Figure 4:**
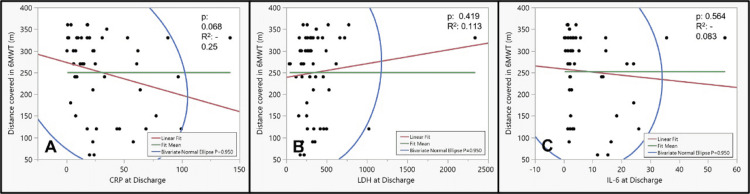
The correlation of inflammatory markers at discharge with six-minute walk distance using fit Y by X bivariate model: (A) CRP, (B) LDH, and (C) IL-6 6MWT: Six-minute walk test; CRP: C-reactive protein; LDH: Lactate dehydrogenase; IL-6: Interleukin-6.

**Figure 5:**
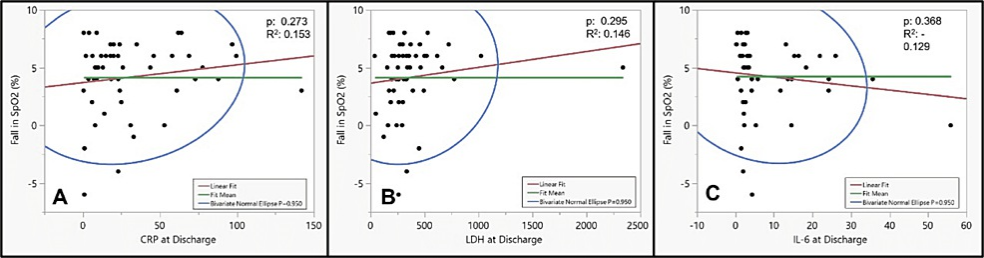
The correlation of inflammatory markers at discharge with fall in SpO2 during six-minute walk test using fit Y by X bivariate model: (A) CRP, (B) LDH, and (C) IL-6 CRP: C-reactive protein; LDH: Lactate dehydrogenase; IL-6: Interleukin-6.

On fit Y by X analysis (logistic fit), none of the inflammatory markers correlated well with the 6MWT parameters (Figures 4, 5). 

The differences in different study variables among the patients who passed or failed 6MWT are enumerated in Table 2.

**Table 2:** Differences in inflammatory markers at discharge, hospitalization characteristics, and 6MWT variables between subjects who passed and failed the 6MWT SD: Standard deviation; Min: Minimum; Max: Maximum; 6MWT: Six-minute walk test; IL-6: Interleukin-6; CRP: C-reactive protein; LDH: Lactate dehydrogenase; O_2_: Oxygen; ∆SpO_2_: Change in SpO_2_ from the beginning to the end of the 6MWT.

	6MWT Failed					6MWT Passed						
Parameters	Mean	Min	Max	SD	Median	Mean	Min	Max		SD	Median	p-value
Inflammatory markers at discharge												
CRP at discharge	31.12	0.6	99.61	29.26	18.4	28.155	0.5		142	33.60	23	0.5447
LDH at discharge	440.80	38.96	2339	396.94	334	265.06	49.2		494	106.95	261	0.0203
IL-6 at discharge	7.19	0.25	35.65	9.07	3.09	9.20	0.6		55.89	13.35	3.43	0.6860
Hospitalization characteristics												
O_2_ days	8.87	0	24	6.05	8	6.9	0		21	5.77	6	0.2465
Hospital days	12.54	5	26	5.97	11	10.95	3		24	5.96	10	0.3503
Non-O_2_ days	2.60	1	5	1.08	3	2.75	1		5	0.96	3	0.7021
Measurable variables among 6MWT												
Time walked (Min)	4.66	1	6	1.68	6	5.55	1		6	1.39	6	0.0193
∆SpO_2_ (%)	6	4	8	1.22	6	1.1	-6		3	2.77	2	<0.0001
Change in pulse	16	-44	38	15.22	16	14.7	-3		46	12.60	10	0.2114
Distance covered in 6MWT (m)	239.69	60	360	99.70	270	268.5	60		360	76.31	300	0.6430
Demographic Characteristics												
Age (Years)	53.06	28	85	14.15	56	57	34		84	14.68	59	0.2624

## Discussion

In this single-center study of 53 discharge-ready patients admitted for severe COVID-19 illness, we found that around two-thirds of patients failed 6MWT. Among the inflammatory markers, LDH levels, but not CRP or IL-6 levels, were significantly different between those who passed or failed the 6MWT. However, none of the inflammatory markers correlated with 6MWT parameters on bivariate analysis.

6MWT is a reproducible and validated test for assessing exercise capacity and predicting outcomes, including mortality, in chronic lung diseases like chronic obstructive pulmonary disease (COPD) [9-11]. 6MWD is the most robust variable assessed during 6MWT and correlates best with pulmonary function tests and outcomes. Change in 6MWD is ideally suited for studying the effect of clinical interventions in chronic respiratory diseases [11]. Apart from 6MWD, SPO_2_ fall during 6MWT gives valuable information about the severity of underlying respiratory physiology and correlates well with pulmonary function tests, particularly diffusion capacity [12-14].

Although widely advocated [3-5], exertional desaturation tests have not yet been validated in patients with COVID-19. Preliminary studies have shown good correlations of 6MWD and ΔSPO_2_ with disease severity, diffusion capacity, and lung volumes [2,15,16]. 6MWT has been studied in the pre-hospital setting (to assess the need for hospitalization), hospital setting (to determine the discharge readiness), and post-hospital setting (to determine the impact of post-COVID illness) [3,4,6,15,17,18]. In this study, we assessed the test's application in discharge-ready patients after recovery from severe COVID-19 illness. In another study that evaluated 26 discharge-ready patients, without any chronic pulmonary disease or heart failure, at a university hospital in Denmark, it was shown that 13 (50%) patients had exertional desaturation (SPO_2 _< 90%). They also found that these patients with exertional desaturation perceived less dyspnea compared to patients of idiopathic pulmonary fibrosis with similar exertional desaturation [6]. In our study, 62.26% of patients failed the 6MWT, questioning the aptness of the prevailing discharge guidelines.

We also found that LDH levels were significantly higher in those discharge-ready patients that failed 6MWT than those that did not. A similar finding was not reproducible with other studied markers, i.e., CRP and IL-6. LDH is an established marker of severe COVID-19 pneumonia [19,20]. The rise of serum LDH in patients with severe illness is attributable to three mechanisms. First, apoptosis of cells during viral replication leads to the release of intracellular LDH; second, dysregulated and exaggerated inflammation in these patients leads to the release of LDH from organs and tissues; and lastly, in hypoxic patients, LDH is upregulated because of its role in oxygen homeostasis. Hypoxia leads to increased lactate generation through the glycolytic cycle, and LDH is upregulated in pyruvate fermentation and metabolic regulation of hypoxic response [19,21]. All three mechanisms are likely operational during acute COVID illness; LDH is elevated along with other inflammatory markers. However, after recovery from acute illness, other inflammatory markers would progressively settle down. Higher LDH in discharge-ready patients with exertional hypoxia can be explained by hypoxia-induced upregulation of LDH.

Our study was limited by the small sample size and a control group's absence. We also did not have follow-up data on these patients that could have guided the incorporation of 6MWT in discharge criteria. Longitudinal studies are suggested to validate the role of 6MWT in assessing discharge readiness after recovery from severe COVID-19 illness.

## Conclusions

Around two-thirds of the patients ready for discharge from the hospital after recovery from severe COVID-19 pneumonia had exertional intolerance in the form of significant exercise desaturation or inability to complete a 6MWT. On bivariate analysis, none of the inflammatory markers at discharge correlated with 6MWT parameters. Serum LDH, but not the other inflammatory markers, was significantly elevated in patients who failed 6MWT at the time of discharge. The major implications of the study are the inadequacy of current discharge criteria to identify complete recovery, the requirement of an additional period of convalescence to allow the patient to restore normalcy of respiratory physiology, and the lack of surrogate biomarkers to identify individuals with exertional hypoxia.
